# Soluble adenylyl cyclase links Ca^2+^ entry to Ca^2+^/cAMP-response element binding protein (CREB) activation in vascular smooth muscle

**DOI:** 10.1038/s41598-019-43821-3

**Published:** 2019-05-13

**Authors:** Tony Parker, Kai-Wen Wang, Declan Manning, Caroline Dart

**Affiliations:** Institute of Integrative Biology, University of Liverpool, Liverpool, L69 7ZB United Kingdom

**Keywords:** Cell signalling, Cardiovascular biology

## Abstract

Ca^2+^-transcription coupling controls gene expression patterns that define vascular smooth muscle cell (VSMC) phenotype. Although not well understood this allows normally contractile VSMCs to become proliferative following vessel injury, a process essential for repair but which also contributes to vascular remodelling, atherogenesis and restenosis. Here we show that the Ca^2+^/HCO_3_^−^-sensitive enzyme, soluble adenylyl cyclase (sAC), links Ca^2+^ influx in human coronary artery smooth muscle cells (hCASMCs) to 3′,5′-cyclic adenosine monophosphate (cAMP) generation and phosphorylation of the transcription factor Ca^2+^/cAMP response element binding protein (CREB). Store-operated Ca^2+^ entry (SOCE) into hCASMCs expressing the FRET-based cAMP biosensor H187 induced a rise in cAMP that mirrored cytosolic [Ca^2+^]. SOCE also activated the cAMP effector, protein kinase A (PKA), as determined by the PKA reporter, AKAR4-NES, and induced phosphorylation of vasodilator-stimulated phosphoprotein (VASP) and CREB. Transmembrane adenylyl cyclase inhibition had no effect on the SOCE-induced rise in cAMP, while sAC inhibition abolished SOCE-generated cAMP and significantly reduced SOCE-induced VASP and CREB phosphorylation. This suggests that SOCE in hCASMCs activates sAC which in turn activates the cAMP/PKA/CREB axis. sAC, which is insensitive to G-protein modulation but responsive to Ca^2+^, pH and ATP, may therefore act as an overlooked regulatory node in vascular Ca^2+^-transcription coupling.

## Introduction

In healthy blood vessels, the majority of vascular smooth muscle cells (VSMCs) are quiescent, contractile and proliferate slowly^1^. Quiescent VSMCs change to a synthetically active and proliferative phenotype in response to stimuli such as mechanical stress, growth factors or inflammatory mediators^2^. This plasticity is important for vessel repair at times of vascular injury, but is also a central feature of vascular disease. Proliferation of VSMCs in the vessel wall contributes to atherogenesis, vascular remodelling in pulmonary hypertension, and to restenosis, a narrowing of the vessel lumen following balloon angioplasty and bypass vein grafting^3–5^. The altered pattern of gene expression that drives changes in VSMC phenotype correlates with different patterns of Ca^2+^ signaling^6,7^, although the molecular basis of Ca^2+^-regulated gene expression in VSMCs is not well understood.

Ca^2+^-induced gene expression can be mediated in VSMCs by the transcription factor Ca^2+^/cyclic AMP response element binding protein (CREB)^6^. CREB binds as a dimer to a conserved cyclic AMP response element (CRE) in the promoter of pro-survival genes and activates transcription in response to a variety of diverse extracellular signals^8^. Multiple Ca^2+^-regulated signaling cascades converge to phosphorylate CREB at a critical Ser^133^ residue, which induces recruitment of coactivators and the formation of an active transcriptional complex^9^. Ser^133^ is a substrate for a number of kinases that are either directly or indirectly activated by Ca^2+^, including protein kinase A (PKA), protein kinase C, members of the calcium/calmodulin-dependent kinase (CaMK) family and ribosomal S6 kinase (RSK) −2^6^. Intriguingly, Ca^2+^ signals from different sources trigger different patterns of CRE-regulated VSM gene expression^10^. Voltage-dependent Ca^2+^ influx through L-type Ca^2+^ channels induces CREB phosphorylation in both cultured VSMCs and in intact mouse cerebral arteries^11,12^ and correlates with an increase in transcription of proliferation-associated CRE-containing genes such as *c-fos* and early growth response-1, *egr-1*^10–12^. In agreement with this, cerebral arteries from hypertensive rats are depolarized compared to normotensive animals and have elevated cytosolic Ca^2+^ and increased CREB phosphorylation/c-fos expression, which can be reversed by L-type Ca^2+^ channel inhibition^13^. Ca^2+^ influx in response to depletion of the sarcoplasmic reticulum (SR) Ca^2+^ stores (store-operated Ca^2+^ entry, SOCE) also activates CREB in both cultured VSMCs and intact arteries. Here, CREB activation leads to the transcription of a set of CRE-regulated genes that overlaps, but is distinct from, the genes expressed following L-type Ca^2+^ channel activation^10,14^. Several transient receptor potential (TRP) channels as well as the Ca^2+^ release-activated channel protein 1 (Orai1) and the SR Ca^2+^ sensor stromal interaction molecule 1 (STIM1) are expressed in VSM and may mediate SOCE^15,16^. Orai1 and STIM1 are both upregulated in response to vascular injury^17^ and are also required for the activation of CREB involved in VSMC proliferation^18^. In line with this, STIM1 knockdown decreases SOCE-induced CREB activation in human coronary artery SMCs and suppresses cell growth^19^. STIM2 is also upregulated in pulmonary artery hypertension, which raises basal Ca^2+^ levels and enhances pulmonary artery VSMC proliferation and vascular remodelling at least partially through activation CREB^20^. Indeed, inhibition of Orai-mediated Ca^2+^ entry and the resultant suppression of Ca^2+^-transcription coupling has been suggested to underlie the anti-proliferative effects of stent-coating drug sirolimus (rapamycin) in human arteries^21^.

In cultured VSMCs CREB activation is associated with both pro-^22–25^, and anti-proliferative outcomes^26,27^, and this complex behaviour is also reflected *in vivo*. In rat models, phospho-CREB is elevated in neointimal VSMCs that appear after balloon injury of the carotid artery^28^, and increased phospho-CREB levels correlate with high proliferation rates in cerebral artery following several forms of vessel injury linked with susceptibility to stroke^29^. In contrast, analysis of arteries taken from bovine systemic and pulmonary circulation show a reverse correlation between CREB phosphorylation and proliferation, with phospho-CREB high in quiescent, contractile smooth muscle cells from the healthy vessel’s medial layer^30^. Here, chronic exposure of animals to lower than normal oxygen levels leads to increased VSMC proliferation in pulmonary vessels and decreased levels of both CREB and phospho-CREB^30^. This highlights our incomplete understanding of how different environmental cues are integrated by VSMCs to activate gene transcription.

The current model for PKA-mediated phosphorylation of CREB in VSMCs is that Ca^2+^ entering the cytosol binds to calmodulin (CaM) and that the Ca^2+^/CaM complex then activates Ca^2+^-sensitive transmembrane adenylyl cyclases (tmACs), leading to generation of cyclic AMP and the activation of PKA^8^. An alternative source of cyclic AMP comes from soluble adenylyl cyclase (sAC), a novel enzyme that lacks the two hydrophobic domains that embed tmACs in the membrane and which thus distributes about the cell in regions remote from the plasma membrane^31^. Here, we demonstrate that SOCE activates sAC to generate cyclic AMP and induce phosphorylation of CREB at Ser^133^ in human coronary artery smooth muscle cells. sAC is insensitive to G-protein modulation and forskolin activation, but directly activated by Ca^2+^ (independently of CaM) and HCO_3_^−^ and responds to physiologically relevant ATP concentrations^32^. Since CO_2_ is in equilibrium with HCO_3_^−^ due to the activity of carbonic anhydrases, sAC functions as a CO_2_/HCO_3_^−^, and thus indirect, pH sensor. This ability to sense and integrate fluctuations in intracellular signals suggests sAC may be an important and overlooked regulatory node in the VSMC Ca^2+^-transcription pathway.

## Results

### SOCE increases intracellular cyclic AMP and activates PKA in human coronary artery smooth muscle cells (hCASMCs)

**Figure 1:**
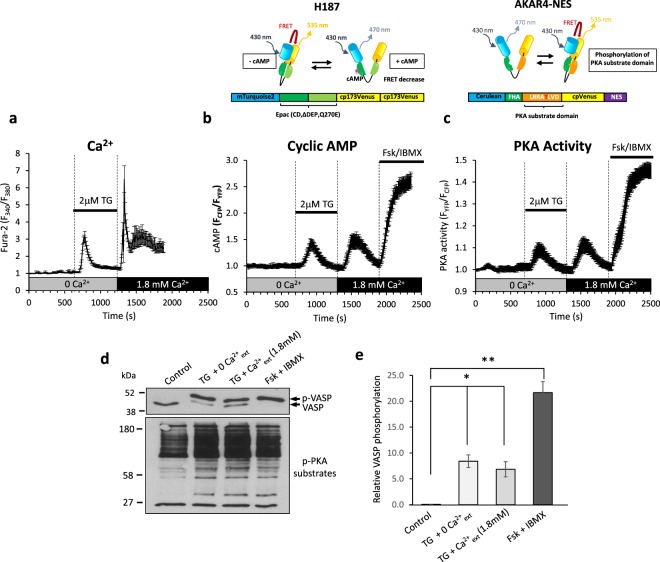
SOCE increases intracellular cyclic AMP and activates PKA in human coronary artery smooth muscle cells (hCASMCs). (**a**) Intracellular Ca^2+^ measurements recorded in fura-2-AM-loaded hCASMCs. Cells were initially bathed in nominally Ca^2+^-free solution for 10 minutes before treatment with thapsigargin (TG, 2 μM). Ca^2+^ (1.8 mM) was then introduced into the extracellular solution and was present hereafter (n = 54 from 4 experimental repeats). (**b**) Intracellular cyclic AMP levels measured in hCASMCs transduced with adenoviruses encoding the cyclic AMP biosensor H187 (upper panel, see Methods for details) and subjected to the same experimental scheme as described in (**a**). At the end of the experiment saturating concentrations of forskolin (20 μM) and IBMX (500 μM) were added to generate maximal cyclic AMP responses (n = 20 cells from 4 experimental repeats). (**c**) PKA activity measured in hCASMCs transduced with adenoviruses encoding the PKA reporter, AKAR4-NES (upper panel, see Methods for details) in response to the experimental scheme described in **a** (n = 19 cells from 4 experimental repeats). All extracellular solutions contain 1% serum. Error bars represent the standard error of the mean. (**d**) Western blot analyses of hCASMC homogenates immunoblotted with anti-VASP (upper panel) and anti-phospho-PKA substrates (lower panel). Before being lysed, cells were exposed to either: vehicle control (0.1% DMSO); thapsigargin (TG, 2 μM) for 5 min in zero extracellular Ca^2+^, TG for 5 min in zero extracellular Ca^2+^ followed by introduction of 1.8 mM extracellular Ca^2+^ for a further 5 min; or forskolin (20 μM) plus IBMX (500 μM) for 5 min. In lysates immunoblotted with anti-VASP, phosphorylated VASP appears as a slower-migrating band. Maximal phosphorylation is seen following exposure to saturating concentrations of forskolin and IBMX. Blots shown representative of 3 experimental repeats. (**e**) Densiometric analysis; the density of the phospho-VASP immunoreactive band relative to the lower (unphosphorylated) VASP band (n = 3, Control vs TG/0Ca^2+^: P = 0.024; Control vs TG/1.8Ca^2+^: P = 0.026; Control vs Fsk/IBMX: P = 0.001; one-way ANOVA with Student Newman Keuls post-hoc test).

To generate store-operated Ca^2+^ entry (SOCE), hCASMC internal Ca^2+^ stores were depleted by bathing fura-2-AM-loaded cells in nominally Ca^2+^-free solution for 10 minutes before addition of the sarcoplasmic reticulum Ca^2+^ ATPase (SERCA) inhibitor, thapsigargin (TG, 2 μM) to the same solution. Ca^2+^ leaks passively from the stores through permanently open leak channels and is subsequently removed from the cytosol either by extrusion across the plasma membrane or reuptake into the stores by SERCA. Inhibition of SERCA thus blocks reuptake and causes a transient rise in intracellular Ca^2+^ as leak Ca^2+^ briefly builds up in the cytosol before being moved out of the cell by plasma membrane Ca^2+^ ATPases (Fig. 1a). Over time this process depletes Ca^2+^ stores. Subsequent addition of Ca^2+^ (1.8 mM) to the extracellular solution induces a rapid rise in cytosolic Ca^2+^ due to SOCE (Fig. 1a). hCASMCs transduced with adenoviruses encoding the FRET-based cyclic AMP biosensor H187 and exposed to the same protocol exhibit changes in intracellular cyclic AMP that essentially mirror the cytosolic Ca^2+^ increase induced by the addition of thapsigargin and by SOCE (Fig. 1b). As a control, saturating concentrations of the transmembrane adenylyl cyclase activator, forskolin, (20 μM) and the pan-phosphodiesterase inhibitor, IBMX, (500 μM) were added at the end of each experiment to generate maximal cyclic AMP responses. These intracellular increases in cyclic AMP activated the major downstream cyclic AMP effector, protein kinase A (PKA). PKA activity was measured in real-time in hCASMCs transduced with adenoviruses encoding the PKA reporter, AKAR4-NES (Fig. 1c). PKA activity was also monitored by immunoblot to determine the phosphorylation status of multiple PKA substrates (PKA substrate antibody detects proteins containing a phospho-Ser/Thr residue within the PKA recognition motif Arg-Arg-X-Ser/Thr) and of the specific PKA substrate, vasodilator-stimulated phosphoprotein (VASP)^33,34^ (Fig. 1d).

### SOCE-generated cyclic AMP is abolished by acute inhibition of soluble adenylyl cyclase

**Figure 2:**
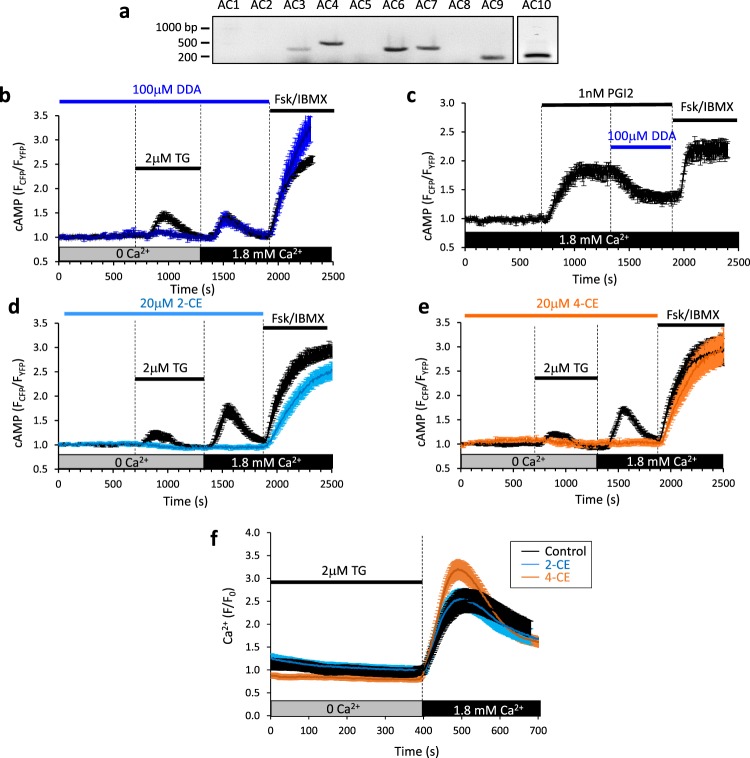
SOCE-generated cyclic AMP is abolished by inhibition of soluble adenylyl cyclase. (**a**) Primers designed to amplify adenylyl cyclase isoforms (1–10) were used to probe hCASMC cDNA. PCR products were separated on a 3% agarose gel. (**b**–**e**) hCASMCs transduced with adenoviruses encoding the cyclic AMP biosensor H187 and subjected to the same experimental scheme as described in Fig. 1. Cells were pre-incubated in either vehicle control (0.1% DMSO; black all traces); the transmembrane adenylyl cyclase inhibitor 2′-5′ dideoxyadenosine (DDA, 100 μM, n = 48 from 4 experimental repeats, (**b**)) or soluble adenylyl cyclase inhibitors 2-hydroxyestradiol (2-CE, 20 μM, n = 29 from 4 experimental repeats (**d**)) or 4-hydroxyestradiol (4-CE, 20 μM, n = 18 from 4 experimental repeats, (**e**)) for at least 10 minutes before addition of thapsigargin (TG, 2 μM). Inhibitors were then present until addition of forskolin (20 μM) and IBMX (500 μM) and the paired controls were conducted on the same day as the test. (**c**) As a positive control, addition of DDA (100 μM) induced a significant reduction in cyclic AMP generated in response to prostacyclin (PGI2; 1 nM, n = 24 from 4 experimental repeats). Error bars represent the standard error of the mean. (**f**) Store-operated Ca^2+^ entry recorded in Fluo-4AM-loaded hCASMCs under control conditions and in the presence of either 2-CE and 4-CE (20 μM). Cells were initially bathed in nominally Ca^2+^-free solution for 10 minutes before treatment with thapsigargin (TG, 2 μM). Ca^2+^ (1.8 mM) was then introduced into the extracellular solution (control, n = 90 cells; 2-CE, n = 65 cells; 4-CE, n = 89 cells). Error bars represent the standard error of the mean.

The Ca^2+^ sensitivity of some isoforms of the enzyme adenylyl cyclase is a well-defined mechanism linking Ca^2+^ influx to the generation of cyclic AMP^35^. Mammals express nine transmembrane isoforms of adenylyl cyclase (tmAC1-9) and one soluble isoform (sAC; AC10), which are differentially regulated by Ca^2+^ ^36^. Of the tmACS, AC5 and AC6 are inhibited by sub-micromolar concentrations of Ca^2+^ while AC1 and AC8, and to a lesser extent AC3, are stimulated by Ca^2+^ via CaM binding^36^. Consistent with other findings^37–39^, reverse transcription-PCR, using cDNA derived from hCASMCs as a template and primers designed to detect AC isoforms 1–10, confirmed the presence of transcripts for AC3, 4, 6, 7, 9 and 10 (Fig. 2a). Since AC3 has been reported to be weakly activated by Ca^2+^/CaM^40^ we tested the effect of acutely inhibiting tmACs on the SOCE-generated cyclic AMP. 2′-5′ dideoxyadenosine (DDA) is a well-characterised cell-permeable pharmacological tool for distinguishing between tmACs and sAC. In whole-cell lysates DDA inhibits tmACs with an IC_50_ of ~10 μM and sAC with an IC_50_ of over 500 μΜ^41^. Preincubation of hCASMCs in DDA (100 μM) significantly reduced the transient increase in cyclic AMP seen upon addition of thapsigargin, but had no effect on SOCE-generated cyclic AMP (Fig. 2b). In parallel control experiments, addition of DDA (100 μM) induced a significant reduction in cyclic AMP generated in response to prostacyclin (PGI2; 1 nM, Fig. 2c). Pre-incubation with the catechol estrogens, 2-hydroxyestradiol (2-CE, 20 μM) or 4-hydroxyestradiol (4-CE, 20 μM), which in cell-based systems are selective sAC inhibitors^41^, completely abolished generation of cyclic AMP in response to both the addition of thapsigargin and SOCE (Fig. 2d,e). Aside from effects on sAC, 2CE and 4CE could potentially block the production of cyclic AMP by inhibiting Ca^2+^ entry into the cell. Since 2CE has previously been shown to inhibit voltage-gated Ca^2+^ influx in pig coronary arteries^42^, we assessed the effects of 2CE and 4CE on SOCE. Exposure of hCASMCs to either 2CE or 4CE did not inhibit Ca^2+^ entry following thapsigargin-induced store depletion and the reintroduction of extracellular Ca^2+^ (Fig. 2f**)**.

### Inhibition of soluble adenylyl cyclase abolishes SOCE-induced VASP phosphorylation

**Figure 3:**
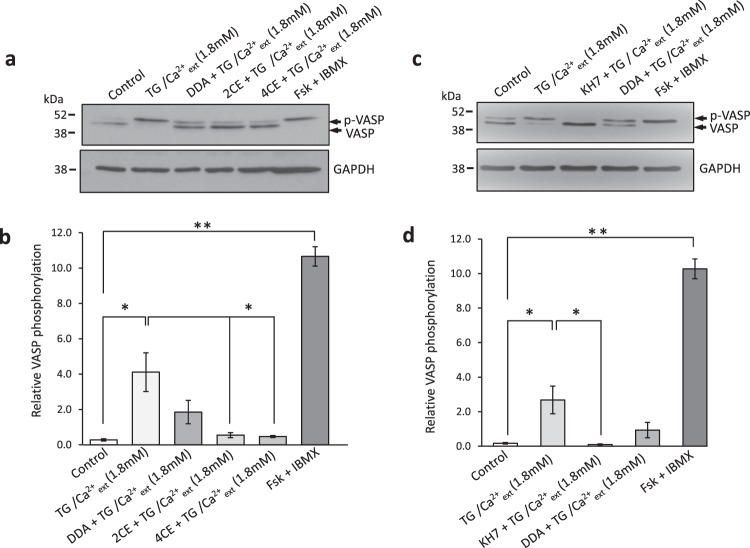
Inhibition of soluble adenylyl cyclase abolishes SOCE-induced VASP phosphorylation. Immunoblot blot analyses of hCASMC homogenates immunoblotted with anti-VASP and anti-GAPDH (loading control). Before being lysed, cells were exposed to either: vehicle control (0.1% DMSO, Lane 1 both blots) or thapsigargin (TG, 2 μM) for 5 min in zero extracellular Ca^2+^ followed by introduction of 1.8 mM extracellular CaCl_2_ for a further 5 min (Lane 2 both blots). (**a**) Cells in Lane 3–5 were exposed to the same protocol as Lane 2, with the exception that they were pre-incubated in either DDA (100 μM; Lane 3), 2-CE (20 μM; Lane 4) or 4-CE (20 μM; Lane 5) for 15 minutes before exposure to TG. (**c**) Cells in Lane 3–4 were exposed to the same protocol as Lane 2, with the exception that they were pre-incubated in either KH7 (20 μM; Lane 3) or DDA (100 μM; Lane 4) for 15 minutes before exposure to TG. Inhibitors were then present throughout the experiment. The final lane in both blots is a positive control where cells were exposed to saturating concentrations of forskolin (20 μM) and IBMX (500 μM) only. Proteins within the homogenates were separated on 10% polyacrylamide-Tris gels. Phosphorylated VASP appears as a slower-migrating band in the upper panel (a,c). Blots shown representative of 3 experimental repeats. (**b**) Densiometric analysis of experiments shown in a; the density of the phospho-VASP immunoreactive band relative to the lower (unphosphorylated) VASP band (n = 3, Control vs TG/1.8Ca^2+^: P = 0.011; TG/1.8Ca^2+^ vs DDA/TG/1.8Ca^2+^: P = 0.892; TG/1.8Ca^2+^ vs 2CE/TG/1.8Ca^2+^: P = 0.030; TG/1.8Ca^2+^ vs 4CE/TG/1.8Ca^2+^: P = 0.026; one-way ANOVA with Student Newman Keuls post-hoc test). (**d**) Densiometric analysis of experiments shown in c; the density of the phospho-VASP immunoreactive band relative to the lower (unphosphorylated) VASP band (n = 3, Control vs TG/1.8Ca^2+^: P = 0.03; TG/1.8Ca^2+^ vs KH7/TG/1.8Ca^2+^: P = 0.037; TG/1.8Ca^2+^ vs DDA/TG/1.8Ca^2+^: P = 0.064 one-way ANOVA with Student Newman Keuls post-hoc test).

In line with our FRET-based studies, pre-incubation of cells with 2CE (20 μM) or 4CE (20 μM) significantly reduced SOCE-induced phosphorylation of VASP (Fig. 3a,b). For these experiments, we exposed hCASMCs to the thapsigargin-induced store depletion/Ca^2+^ reintroduction protocol (Figs 1, 2) in the presence or absence of AC inhibitors, lysed cells and analysed lysates by immunoblot using antibodies against VASP. We also tested the effects of KH7, a selective inhibitor of sAC with intrinsic fluorescence that makes it unsuitable for use in fluorescent microscopy. In whole cell lysates KH7 inhibits sAC with an IC_50_ of 20 μM while having no significant effect on tmAC activity at concentrations up to 1 mM^41^. Pre-incubation with KH7 (20 μM) significantly reduced SOCE-induced VASP phosphorylation (Fig. 3c,d). The effect of DDA (100 μM) on SOCE-induced VASP phosphorylation did not reach significance.

### Cyclic AMP generated by soluble adenylyl cyclase in response to SOCE phosphorylates CREB

**Figure 4:**
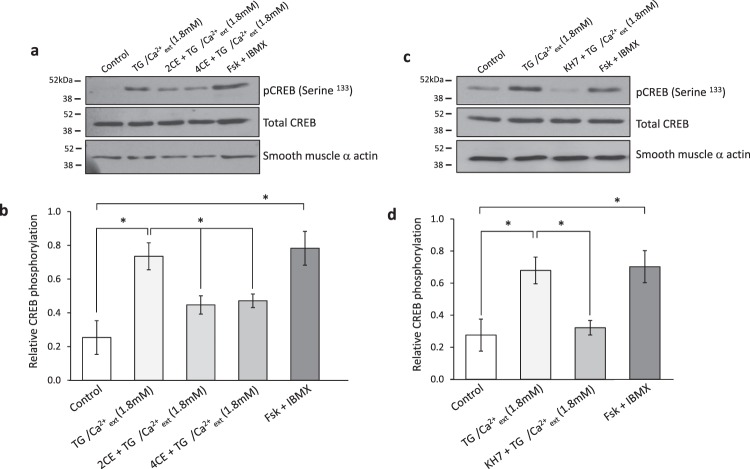
Cyclic AMP generated by sAC in response to SOCE phosphorylates CREB. Immunoblot analyses of hCASMC homogenates immunoblotted with antibodies specific to the Ser^133^ phospho-site on CREB (pCREB), total CREB and smooth muscle α actin (loading control). Before being lysed, cells were exposed to either: vehicle control (0.1% DMSO, Lane 1 both blots) or thapsigargin (TG, 2 μM) for 5 min in zero extracellular Ca^2+^ followed by introduction of 1.8 mM extracellular CaCl_2_ for a further 5 min (Lane 2 both blots). (**a**) Cells in Lane 3–4 were exposed to the same protocol as Lane 2, with the exception that they were pre-incubated in 2-CE (20 μM; Lane 3) or 4-CE (20 μM; Lane 4) for 15 minutes before exposure to TG. (**c**) Cells in Lane 3 were exposed to the same protocol as Lane 2, with the exception that they were pre-incubated in KH7 (20 μM) for 15 minutes before exposure to TG. Inhibitors were then present throughout the experiment. The final lane in both blots is a positive control where cells were exposed to saturating concentrations of forskolin (20 μM) and IBMX (500 μM) only. Proteins within the homogenates were separated on 10% polyacrylamide-Tris gels. Blots shown representative of at least 3 experimental repeats. (**b**) Densiometric analysis of experiments shown in a; the relative density of the phospho-CREB immunoreactive band (n = 3, Control vs TG/1.8Ca^2+^: P = 0.010; TG/1.8Ca^2+^ vs 2CE/TG/1.8Ca^2+^: P = 0.048; TG/1.8Ca^2+^ vs 4CE/TG/1.8Ca^2+^: P = 0.023; one-way ANOVA with Student Newman Keuls post-hoc test). (**d**) Densiometric analysis of experiments shown in c; the relative density of the phospho-CREB immunoreactive band (n = 6, Control vs TG/1.8Ca^2+^: P = 0.018; TG/1.8Ca^2+^ vs KH7/TG/1.8Ca^2+^: P = 0.014; one-way ANOVA with Student Newman Keuls post-hoc test).

To assess the downstream consequences of SOCE-generated cyclic AMP, we also analysed hCASMC lysates by immunoblot using antibodies specific to CREB’s Ser^133^ phospho-site (Fig. 4). Figure 4a,c (lanes 1–2) shows that thapsigargin-induced store depletion followed by addition of Ca^2+^ (1.8 mM) to the extracellular solution induces significant phosphorylation of CREB at Ser ^133^. Pre-incubation with the catechol estrogens 2CE and 4CE (Fig. 4a,b), or KH7 (Fig. 4c,d), significantly reduced SOCE-induced CREB phosphorylation.

## Discussion

Our data suggest that Ca^2+^ influx in response to hCASMC store depletion activates the Ca^2+^ and HCO_3_^−^-sensitive enzyme, sAC, which generates cyclic AMP to activate PKA/CREB. Intracellular cyclic AMP levels in hCASMCs largely mirrored the changes in cytosolic free Ca^2+^ seen during thapsigargin-induced store depletion and subsequent SOCE. Inhibition of tmACs significantly reduced the intracellular increases in cyclic AMP seen during thapsigargin-induced store depletion, but had no significant effect on SOCE-generated cyclic AMP. In agreement with other studies, we found no evidence for expression of the Ca^2+^-activated AC1 or AC8 in these cells, although we did find transcripts for AC3, which is weakly activated by Ca^2+^ (half-maximal activation 5 μM)^40^. Acute inhibition of sAC abolished the intracellular increases in cyclic AMP seen during thapsigargin-induced store depletion and subsequent SOCE, suggesting that activation of sAC is a prerequisite for cyclic AMP generation via both processes. To our knowledge, SOCE has not previously been reported to activate sAC, although Ca^2+^ influx through voltage-gated Ca^2+^ channels activates sAC in insulinoma cells^43^.

By using biosensors to monitor real-time changes in the activity of the major cyclic AMP effector, PKA, our data also show that SOCE/sAC-generated cyclic AMP activates PKA. This induces phosphorylation of a number of PKA substrates within the cell, including VASP and the transcription factor, CREB. Previous studies have shown that Ca^2+^ influx through store-operated channels activates CREB and induces transcription of a distinct set of genes in VSMCs^10^, but the pool of cyclic AMP responsible for PKA-mediated CREB activation was believed to be generated exclusively by tmACs. More recent work shows that CREB can mediate both pro- and anti-mitogenic responses in VSMCs dependent upon different modes of activation^44^. Activation of CREB by cyclic AMP generated by either forskolin or agonists at G protein-coupled receptors (both of which exclusively activate tmACs) is independent of Ser^133^ phosphorylation but dependent on nuclear translocation of CREB Regulated Transcription Coactivators (CRTCs)^44,45^. This form of CREB activation is anti-proliferative. In contrast, stimulation of VSMCs by mitogens significantly increases CREB activity via Ser^133^ phosphorylation but not CRTC activation. CREB activated by this mechanism induces pro-mitogenic responses since CREB silencing under these circumstances inhibits basal and growth factor-induced proliferation^44^. Our studies, which were conducted in the presence of serum and show Ser^133^ phosphorylation, suggest that sAC-generated cyclic AMP may contribute to this latter pro-proliferative pathway.

Compared to the relatively well-studied tmACs, our knowledge of the role of sAC in the vasculature is limited. Although widely expressed throughout the cardiovascular system^46^, the only previously reported role for vascular sAC involves activation by oxidative stress to promote the intrinsic apoptotic pathway^47^. Similar roles in apoptotic signaling have been described in coronary endothelial cells^48^ and cardiomyocytes^49^. In cardiomyocytes there is evidence of roles for sAC outside of death pathways in the development of cardiac hypertrophy^50^, and in matching energy production to nutrient levels in mitochondria^51^. Here, CO_2_/HCO_3_^−^ generated by the citric acid cycle activates sAC localized in the matrix to produce cyclic AMP, which mediates a PKA-dependent increase in the activity of the electron transport chain and oxidative phosphorylation^51,52^. A source of cyclic AMP that is distinct from the G-protein-responsive plasma membrane-localized tmACs is likely to be an important general feature in signaling. Cyclic AMP has a limited diffusion distance largely due to the action of phosphodiesterases that restrict its spread throughout the cell to ensure the fidelity of signal transfer to intended targets^53^. sAC is able to locate to several intracellular compartments, including the cytosol, the nucleus^54^, and the mitochondria^51,55^. Indeed, in skin cells, sAC localizes to the nucleus where it complexes with CREB^56^. Zippin *et al*. demonstrated the existence of a functional signaling complex consisting of sAC, the PKA holoenzyme and CREB in the nucleus of human cell lines and in a subpopulation of rat liver cells^54^. This complex ensures rapid CREB activation in response to changes in HCO_3_^−^ and was distinct from the slower hormone or neurotransmitter CREB activation via tmACs.

In conclusion, our data suggest that in hCASMCs SOCE activates the Ca^2+^ and HCO_3_^−^-sensitive sAC, which generates cyclic AMP to activate the PKA/CREB axis. Being located away from the plasma membrane may allow sAC access to pools activators and effectors that are distinct from the membrane-confined tmAC microdomains. This coupled with its ability to respond rapidly to changes in the intracellular environment suggests it may act as a regulatory point in VSMC Ca^2+^-transcription coupling. Although the functional consequences of sAC-induced CREB phosphorylation are undetermined, recent evidence suggests it may link to pro-mitogenic responses in VSMCs^44^. A single gene encodes sAC, which is widely expressed in mammalian tissues^31^. Alternative splicing generates multiple sAC isoforms^57^, suggesting that VSMC sAC may warrant investigation as a potentially novel drug target for proliferative vascular disease. It will also be important to determine if specific subsets of phosphodiesterases control the dispersal of sAC-generated cyclic AMP in VSMCs as these ‘druggable’ targets may offer an additional route to manipulate the signaling pathway.

## Materials and Methods

### Chemicals

2′-5′dideoxyadenosine (DDA) and thapsigarin were purchased from Merck Millipore (Watford, UK). (6)-2-(1*H*-benzimidazol-2-ylthio)propanoic acid 2-[(5-bromo-2-hydroxyphenyl)methylene]hydrazide (KH7) and forskolin were purchased from Tocris Bioscience (Abingdon, UK). Fura-2-AM and Fluo-4 AM were purchased from ThermoFisher Scientific (Paisley, UK). All other chemicals, including 2-hydroxyestradiol (2-CE), 4-hydroxyestradiol (4-CE), and 3-isobutyl-1-methylxanthine (IBMX) were purchased from Sigma Aldrich (Gillingham, UK).

### Cell culture

Human coronary artery smooth muscle cells (hCASMCs; Promocell, Heidelberg, Germany) were cultured in smooth muscle cell growth medium 2 containing 5% (v/v) foetal calf serum supplemented with basic fibroblast growth factor (2 ng mL^−1^), epidermal growth factor (0.5 ng mL^−1^) and insulin (5 μg mL^−1^; all Promocell). Cells between passages 6–12 were cultured in a humidified incubator at 37 °C and 5% CO_2_ atmosphere, with culture media changed every 2–3 days. Cells were routinely sub-cultured at 70–80% confluency.

### Adenovirus transduction

Adenoviruses encoding H187 [AD(RGD)-CMV-H187] and AKAR4 [AD(RGD)-CMV-AKAR4-NES] were cloned, packaged, amplified and titrated by Vector Biosystems Inc (Malvern, PA, USA). For FRET-based imaging studies, viral particles of either AD(RGD)-CMV-H187 or AD(RGD)-CMV-AKAR4-NES in a solution containing Dulbecco’s Modified Eagle Medium (DMEM; Gibco ThermoFisher Scientific), 2% (w/v) bovine serum albumin (BSA; Invitrogen, ThermoFisher Scientific) and 2.5% (v/v) glycerol, were added directly to hCASMC suspensions at 10 MOI (multiplicity of infection). Cells were then seeded onto glass-bottomed culture dishes (Mattek Inc., Bratislava, Slovakia) for imaging 24 or 48 hours later.

### Förster resonance energy transfer (FRET)-based biosensors and fluorescence imaging

Real-time cyclic AMP changes were measured in live hCASMCs transduced with adenoviruses encoding the biosensor H187 (mTurquoise2Δ-Epac(CD,ΔDEP,Q270E)-tdcp173Venus; plasmid used for adenoviral construction a gift from Kees Jalink, Univ of Amsterdam, Netherlands)^58^. This sensor consists of the cAMP effector Epac1 fused to a cyan fluorescent protein (CFP) donor (mTurquoise2) and an optimized yellow fluorescent protein (YFP) acceptor (a tandem of the circular permutated version of Venus, cp173Venus). Binding of cAMP causes a conformational change resulting in a loss of energy transfer between the two fluorophores that can be detected as an increase in the CFP/YFP fluorescence ratio. To prevent construct enzymatic activity affecting downstream effectors, Epac has been rendered catalytically dead (CD) and a membrane-targeting domain is deleted (ΔDEPmutant) to allow measurement of cAMP in the bulk cytosol. Real-time PKA catalytic activity was measured in hCASMCs using the FRET-based PKA reporter A-Kinase Activity Reporter-Nuclear Export Signal, AKAR4-NES^59^ (a gift from Jin Zhang, John Hopkins University, Baltimore, USA). AKAR4 contains a PKA substrate and a phospho-amino acid binding domain (PAABD) between the FRET pair Cerulean and Venus. The PAABD binds to the PKA substrate when it is phosphorylated by active PKA, causing a decrease in distance between the fluorophores and an increase in FRET. Prior to each experiment, H187 and AKAR4-expressing hCASMCs were rinsed twice in a Na^+^-HEPES-based solution containing (mM): 140 NaCl, 4.7 KCl, 1.13 MgCl_2_, 10 HEPES, and 10 glucose (all purchased from Sigma Aldrich); adjusted to pH 7.4 (referred to as HEPES solution hereafter). This HEPES solution was also supplemented with 1% (v/v) foetal calf serum (0.4 ng mL^−1^), epidermal growth factor (0.1 ng mL^−1^), insulin (1 μg mL^−1^) and 1.8 mM CaCl_2_ unless stated otherwise. All test solutions, including controls, were made using this HEPES solution.

Cells were imaged using an Olympus IX71 based inverted imaging system (Till Photonics GmbH, Germany). Excitation of fluorescence was provided by a 150 W xenon high stability lamp and a polychrome V monochromator wavelength control (Till Photonics GmbH, Germany). Emission light was collected using band or high pass filters and sent to an iXon DV885 cooled EM CCD camera (Andor technology, UK). Camera binning was set to 2 both horizontally and vertically which provided 512 × 512 pixel points per image. TillVision software (Till Photonics, Germany) was used to control the parameters of image acquisition and selection of regions of interest, and allowed for the measurement of fluorescence while the experiment was in progress. H187 and AKAR4-expressing hCASMCs were both excited at 405 nm, close to the excitation maximum of the donor, CFP. Emissions of both CFP and YFP fluorescence were measured simultaneously using an Optical Insights Dual-View Filter cube with D480 + 30 m and D535 + 40 m Filter Sets, (BioVision Technologies, USA) which only allows emission wavelengths of 480 nm and 535 nm to reach the camera. During each experiment non-fluorescent cells or regions containing no cells were used as background fluorescence from which the reading was subtracted.

### Ca^2+^ fluorimetry

hCASMCs were incubated in 5 µM of the ratiometric Ca^2+^ dye Fura-2 AM dissolved in HEPES solution for 1 hour at room temperature in the dark. Fura-2 AM-loaded cells were then rinsed twice with HEPES solution, before being placed into the perfusion chamber for live cell imaging using the Olympus IX71 based inverted imaging system (above). The cell was illuminated alternately with 340 and 380 nm light (bandpass 5 nm) and the emission signal was detected at 510 nm (40 nm bandpass). Temporal change in photon counts was measured, backgrounds were subtracted, and the complete ratio was obtained at 50 Hz. As in FRET experiments, cells that were not fluorescent or regions containing no cells were used as background from which the reading was subtracted. For indicated experiments, cells were incubated with 5 µM Fluo-4 AM in glass-bottomed dishes (Ibidi GmbH, Germany), washed as described above and imaged using a Zeiss LSM 710 confocal microscope. Cells were excited with 488 nm light and the emission signal was detected within a range of 493–622 nm.

### SDS-PAGE and Immunoblotting

hCASMCs were seeded onto 6-well plates and allowed to grow at 37 °C and 5% CO_2_ atmosphere until 70–80% confluence. Cells were then incubated in vehicle control (0.1% DMSO) or incubated in zero extracellular Ca^2+^ for 5 mins before being treated with thapsigargin (TG, 2 μM) for 5 min in zero extracellular Ca^2+^ followed by introduction of 1.8 mM extracellular CaCl_2_ for a further 5 min. As a positive control cells were exposed to saturating concentrations of forskolin (20 μM) and IBMX (500 μM) only. In some experiments, cells were incubated in the tmAC inhibitor DDA (100 μM) or the sAC inhibitors KH7 or 2CE or 4CE (20 μM) for at least 15 minutes before TG treatment. Inhibitors were then present throughout the experiment. The solution used for all treatments was the same supplemented HEPES solution used for fluorescent imaging. Following each treatment, cells were then lysed in ice-cold radioimmunoprecipitation assay (RIPA) buffer containing 1% (v/v) protease inhibitor cocktail and 1x PhosSTOP phosphatase inhibitor cocktail (both Sigma-Aldrich). The resultant lysates were rotated for 15 minutes at 4 °C and then centrifuged at 18,220 × *g* for 15 minutes at 4 °C. Resultant supernatants were mixed 1:3 (v/v) with 4x sodium dodecyl sulfate-polyacrylamide (SDS) sample buffer, before heating to 98 °C for 10 minutes. Protein concentrations were quantified using a Pierce^TM^ BCA Protein Assay Kit (Thermofisher Scientific). Samples were kept at −20 °C until use. Proteins within the lysates were resolved by SDS-polyacrylamide gel electrophoresis (SDS-PAGE) on 10–15% polyacrylamide gels and transferred electrophoretically onto nitrocellulose membranes (Hybond ECL, GE Healthcare). Immunoblotting was performed as previously described^60^.

### Antibodies

For immunoblotting the primary antibodies used were against: total CREB, vasodilator-stimulated phosphoprotein (VASP) and phospho-PKA substrates (RRXS*/T*); all purchased from Cell Signaling Technologies (Hitchin, UK) and used at a dilution of 1:1000. Anti-smooth muscle *α* actin (dilution 1:10,000) was from Sigma-Aldrich. Anti-phospho-CREB (Ser^133^, dilution 1:1000) and anti-GAPDH (1: 2000) were purchased from Abcam (Cambridge, UK). The secondary antibodies used were: anti-mouse IgG (H + L) horseradish peroxidase (HRP) conjugated polyclonal antibody and anti-rabbit IgG (H + L) HRP conjugated polyclonal antibody (Stratech Scientific Ltd., Newmarket, U.K.), both used at a dilution of 1:5000.

### Total RNA extraction and reverse transcription

Total RNA extraction and reverse transcription was carried out as previously described^61^.

### Polymerase chain reaction (PCR)

Touchdown PCR was carried out as previously described^61^. Primers used to amplify specific adenylyl cyclase isoforms were previously published^62^. Products were analysed by running on a 3% agarose gel containing Midori Green (1:10,000; GC Biotech) for ~1 hour at 80 V. Bands were excised under ultraviolet light and products purified using a QIAquick Gel Extraction Kit (Qiagen) according to manufacturer’s instructions. All products were verified by sequencing (GATC Biotech, Germany).

### Statistical analysis

Statistical tests stated throughout. Results are expressed as the mean ± standard error of mean. For real-time Ca^2+^/cyclic AMP measurements, n numbers indicate number of cells from at least 3 experimental repeats conducted on separate days. For immunoblots n numbers indicate number of experimental repeats.

## Data Availability

The datasets generated during and/or analysed during the current study are available from the corresponding author on reasonable request.
